# Variability of Gross Tumor Volume in Nasopharyngeal Carcinoma Using ^11^C-Choline and ^18^F-FDG PET/CT

**DOI:** 10.1371/journal.pone.0131801

**Published:** 2015-07-10

**Authors:** Jun Jiang, Hubing Wu, Meiyan Huang, Yao Wu, Quanshi Wang, Jianqi Zhao, Wei Yang, Wufan Chen, Qianjin Feng

**Affiliations:** 1 School of Biomedical Engineering, Southern Medical University, Guangzhou, China; 2 Department of PET Center, Nanfang Hospital, Guangzhou, China; Central South University, CHINA

## Abstract

This study was conducted to evaluate the variability of gross tumor volume (GTV) using ^11^C-Choline and ^18^F-FDG PET/CT images for nasopharyngeal carcinomas boundary definition. Assessment consisted of inter-observer and inter-modality variation analysis. Four radiation oncologists were invited to manually contour GTV by using PET/CT fusion obtained from a cohort of 12 patients with nasopharyngeal carcinoma (NPC) and who underwent both ^11^C-Choline and ^18^F-FDG scans. Student’s paired-sample *t*-test was performed for analyzing inter-observer and inter-modality variability. Semi-automatic segmentation methods, including thresholding and region growing, were also validated against the manual contouring of the two types of PET images. We observed no significant variation in the results obtained by different oncologists in terms of the same type of PET/CT volumes. Choline fusion volumes were significantly larger than the FDG volumes (p < 0.0001, mean ± SD = 18.21 ± 8.19). While significantly consistent results were obtained between the oncologists and the standard references in Choline volumes compared with those in FDG volumes (p = 0.0025). Simple semi-automatic delineation methods indicated that ^11^C-Choline PET images could provide better results than FDG volumes (p = 0.076, CI = [–0.29, 0.025]). ^11^C-Choline PET/CT may be more advantageous in GTV delineation for the radiotherapy of NPC than ^18^F-FDG. Phantom simulations and clinical trials should be conducted to prove the possible improvement of the treatment outcome.

## Introduction

The incidence of nasopharyngeal carcinoma (NPC) is higher in southern China than in other countries. To treat this disease effectively, radiotherapy treatment (RT) is recommended by oncologists.

In clinical practice of NPC treatment, MR images are usually used as references to CT images to determine gross tumor volume (GTV) during RT [1]. Registration algorithms have also been developed to deal with alignments of MR and CT images. In theory, visual and alignment errors are inevitably presented when the two modalities are combined. PET/CT scan, which is recommended for NPC diagnosis, provides inherent alignment of anatomical (CT) and functional (PET) information. The PET/CT fused images are also used for GTV delineation and potentially decline inter-observer variability in manual delineation [2–4].

As a new modality for NPC diagnoses, PET is also used as a tool in target definition for patients with non-small-cell lung cancer, head and neck cancer, and rectal cancer during RT planning [5]. Some guidelines have also been developed. Commonly used methods for tumor outlining include qualitative visual method [6], GTV 2.5 standardized uptake value (SUV) units [7], linear adaptive SUV threshold function method [8], and GTV 40% of local maximal uptake value method [9]. These methods were all based on ^18^F-FDG PET images, and none of them directly aimed to delineate GTV for advanced NPC.

**Fig 1:**
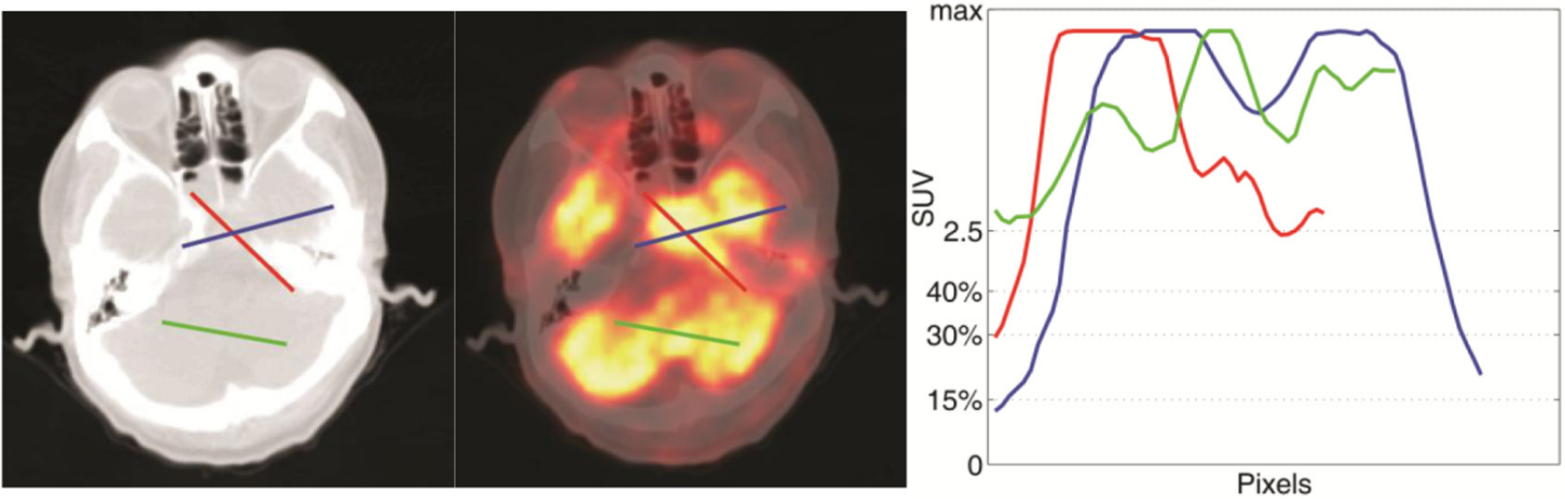
SUV of tumor and surrounding tissuse. On the left side of the figure, two lines are drawn along axial (blue) and radial (red) directions of the lesions. The SUV under the two lines are sampled and shown on the right. SUV_15%_, SUV_30%_, SUV_40%_, SUV_2.5_ baselines are shown along the Y axis. SUV of lesion and normal tissues reveals many overlapping regions.

As we know, intracranial and skull base invasion are frequently observed in patients with locally advanced NPC. Since the SUV of brain tissues are comparable to tumors, threshold method seems inadequate for target contouring using only ^18^F-FDG PET images [Fig 1]. This circumstanceis also accounted for the failure of manual contouring, because ^18^F-FDG PET can not provide sufficient distinction between lesions and normal tissues for advanced NPC. Considering that ^11^C-Choline is commonly used as a tumor-imaging agent in brain tumor diagnosis [10], we conducted a visual evaluation of ^11^C-Choline-PET and ^18^F-FDG-PET for advanced NPC diagnosis. The results suggested that ^11^C-Choline PET/CT may be superior to ^18^F-FDG PET/CT in terms of determining GTV in patients with locally advanced NPC.

Only few studies have been proposed to evaluate the precision of GTV delineation with ^11^C-Choline PET for NPC. In the current study, GTV delineation results were quantitatively evaluated using the two kinds of PET volumes with different segmentation methods.

## Methods

### Ethics Statement

This study was approved by the Ethics Committees of Nanfang Hospital. Patient records/information was anonymized and de-identified prior to analysis.

### Image Acquisition

Examinations were performed using a Discovery LS PET/CT scanner (GE Healthcare) in the Department of PET Center, xx Hospital. A total of 15 patients voluntarily participated in our research from December 2007 to May 2009. The scanning conditions were detailed in our previous study [11]. Patients underwent FDG PET scan before Choline PET scan, and the longest time interval between the two scans was 14 d. Each patient was instructed to undergo fasting for at least 6 h before tracer injection and to minimize any talking, chewing, swallowing, or head movement during image acquisition.

We collected 15 PET/CT scans. The entire study was processed using the original image volume. Ten of the patients were newly diagnosed, and five exhibited recurrent locally advanced NPC. According to pathological examination results, 14 cases manifested non-keratinized undifferentiated carcinoma and 1 case exhibited well-differentiated squamous carcinoma. Intracranial and skull base invasion was diagnosed and confirmed by contrast-enhanced MRI or CT. Original MR and CT DICOM images were also collected as contouring standard GTV reference. Approximately 41.7% (5/12) of the subjects were transferred to different hospitals. To save their data, we contoured their standard GTVs by using fused PET/CT images. However, 41.7% (5/12) subjects’ image data were lost because of hospital transferring, and their standard GTVs were contoured by using fused PET/CT images.

### Image Process

PET volumes (4 mm × 4 mm × 4.25 mm) were interpolated to the same resolution as CT volumes (0.98 mm × 0.98 mm ×2 mm), and the two modalities were aligned according to the physical position information stored in DICOM files. If the volume was misaligned using this method, a conspicuous head movement possibly occurred during data acquisition. Therefore, this volume was excluded from our data set. We selected 12 image volumes with good alignment and imaging quality in our study, and each case contained ^18^F-FDG and ^11^C-Choline images. Patients’ characteristics, time interval, and time sequence of the two PET/CT scans are summarized in Table 1 (Patient numbers in this study correspond to those listed in Table 1 in our previous study [11]).

**Table 1:** Clinical data of patients with NPC.

Pt. No.	Scanning Date*		PET scan Interval(days)	GTV Confirmation		Reference GTV(cm^3^)	
	FDG	CH		Type	Date*	FDG	CH
**1**	08–23	08–25	2	MRI (CE)	07–28	29.18	41.20
**2**	06–30	07–02	2	MRI (CE)	06–16	18.09	36.38
**3**	06–23	06–25	2	MRI (CE)	06–24	19.90	39.83
**5**	04–13	04–14	1	CT (CE)	03–15	45.41	57.11
**6**	04–02	04–03	1	-	03–02	69.51	104.03
**7**	04–02	04–03	1	-	03–18	22.15	42.28
**8**	03–06	03–09	3	MRI (CE)	03–04	41.86	58.44
**9**	01–05	01–06	3	MRI (CE)	12–08	72.58	99.98
**10**	08–22	08–25	3	-	07–25	43.87	49.96
**11**	07–20	07–20	0	-	07–18	36.04	63.20
**12**	08–15	08–29	14	-	06–18	22.59	35.69
**14**	08–25	08–27	2	MRI (CE)	07–14	54.15	65.70

Abbreviation: Pt. No. = Patient Number

-: MRI or CT data missing

*: Scanning date are formated as month-date

Based on the information stored in the image header, body weight (bw) SUVs were calculated according to a previously described method [12]. However, we could not determine whether or not the patients included were affected by primary or recurrent carcinoma, because tumor metastasis and growth of NPC are inherently unpredictable. Our GTV evaluation did not consider the isolated lumps in the neck; instead, we focused only on the primary tumor in the head to simplify the complicated circumstance.

### Image Analysis

The standard reference of GTV was confirmed as ground truth for image analysis. Image analysis was conducted using two categories of volumetric comparison: inter-observer variation (across oncologists within a modality) and inter-modality variation (between FDG and Choline PET/CT fusion for each oncologist and on average). The two categories were analysed with a mean significance test (category-dependent) or an overlap analysis. The significance threshold was set at *α* = 0.05. “Near” significance or “tendency” was noted for 0.05 < *α* < 0.10.

### Standard Reference of GTV

The contouring protocol was performed according to Hani [13]. For the first round, two oncologists with at least four years of clinical experience were invited to contour the GTV of the image volumes respectively. Available enhanced MR or CT was used as complementary images. While, for the second round, oncologists collaborated on contouring GTV. To establish the standard reference of GTV for each type of PET/CT fusion, we compared the scope and border of their contouring results, and recorded any discrepancy between the two GTVs. Oncologists reached a consensus of the standard reference of GTV by deliberating their contouring results [Table 1].

### Inter-observer and Inter-modality Variations

Four oncologists, indexed as A, B, C, and D (with one, two, two, and three years of clinical experiences, respectively), were asked to manually contour the GTV of the 12 image volumes separately. The contouring was implemented frame by frame on both FDG and Choline PET/CT fused images by using the distinct halo described by Hani [13]. To catch apparent border in CT images, oncologists were allowed to turn the view window and transparency freely as they investigate in their respective workstations. All of their contouring results were saved for further analysis and evaluation.

We assessed the inter-observer variation in FDG and Choline PET/CT fusions with the GTV obtained by the four oncologists. Volume differences cannot comprehensively reveal the disparities between contouring results and ground truth, because tumors with same volume can differ in shape or disease sites. To compare the GTV overlap degrees, we introduced the dice similarity coefficient (DSC), which is a commonly used metric in the evaluation of image segmentation [3, 14].

For each result, the false positive (FP), false negative (FN), true positive (TP), and true negative (TN) counts were calculated. These four metrics are defined as follows:

$$TP=R\cap T;TN=\bar{R\cup T};FP=R\cap \bar{T};FN=\bar{R}\cap T$$ (1)

where *T* is the voxel set inside the ground truth; *R* is the voxel set inside the contouring result. From these values, DSC can then be computed:

$$DSC=\frac{2*TP}{\left(FP+TP\right)+\left(TP+FN\right)}$$ (2)

High DSC indicated a high voxel correlation between contouring result and ground truth and vice versa.

Inter-modality variation in the results obtained by each oncologist was also analysed by using Student’s paired-sample *t*-test and a DSC pairwise comparison. Average inter-modality variation was measured by calculating the average of the volumes obtained by the oncologists for each modality and by comparing these results using Student’s paired-sample *t*-test.

### Semi-automatic Evaluation

Manual contouring results are susceptible to subjective factors, such as view window. Some automatic and semi-automatic methods have been proven to be objective because these methods didn’t need human interaction and such methods were not very sensitive to human initialization. To minimize subjective errors, we investigated the performance of two commonly used semi-automatic, threshold-based, and region-growing (RG) methods, on contouring NPC tumor in ^18^F-FDG and ^11^C-Choline PET images. SUV_15%_, SUV_30%_, SUV_40%_, and RG contouring results were compared with ground truth results [15–17]. Inter-modality variations were analysed again as previously conducted to the manual contouring results.

## Results

Using the contouring protocol described by Hani [13], the four oncologists finished their GTV delineation in one month. Contouring was based on fused PET/CT images because the MR images were incomplete. Oncologist A was instructed to re-contour GTV because of a misunderstanding of the contouri ng protocol. Oncologist B was directed to modify the volumes to eliminate lymph node involvement, which was not included in the GTV for the purposes of the study.

The GTV standard references (listed in Table 1) of ^11^C-Choline volumes were significantly larger than their FDG volumes (p < 0.0001, mean ± SD = 18.21 ± 8.19). The mean results of the inter-observer and inter-modality DSC comparison using the manual contouring method are listed in Table 2. Significantly consistent results were detected between oncologists and the standard references in the Choline and FDG volumes (p = 0.0025). Hence, conspicuous improvement of decreasing inter-observer variation was reflected. Significant consistency also occurred between the pairs of oncologists in the two types of PET volumes (p = 0.0001). No significant difference was observed between the results of pairs of oncologists in the same type of PET volumes.

**Table 2:** Results of inter-observer and inter-modality DSC analysis for average PET/CT fusion volumes.

Mod.	Observer	A	B	C	Ref.
**FDG**	A				0.77
	B	0.75			0.81
	C	0.71	0.82		0.83
	D	0.78	0.76	0.80	0.85
**CH**	A				0.92
	B	0.86			0.95
	C	0.88	0.92		0.92
	D	0.91	0.89	0.89	0.97

Abbreviation: Mod. = Modality; Ref. = Standard GTV reference.

The mean results of the inter-modality DSC comparison with semi-automatic methods were listed in Table 3. Semi-automatic methods tend to provide better result in ^11^C-Choline volumes than in FDG volumes (p = 0.076, CI = [–0.29, 0.025]).

**Table 3:** Results of inter-modality DSC analysis of semi-automatic methods.

Method	FDG vs. Ref.	CH vs. Ref.
**SUV**_**15%**_	0.28	0.38
**SUV**_**30%**_	0.35	0.42
**SUV**_**40%**_	0.36	0.44
**RG**	0.34	0.62

Abbreviation: Ref. = Standard GTV reference.

**Fig 2:**
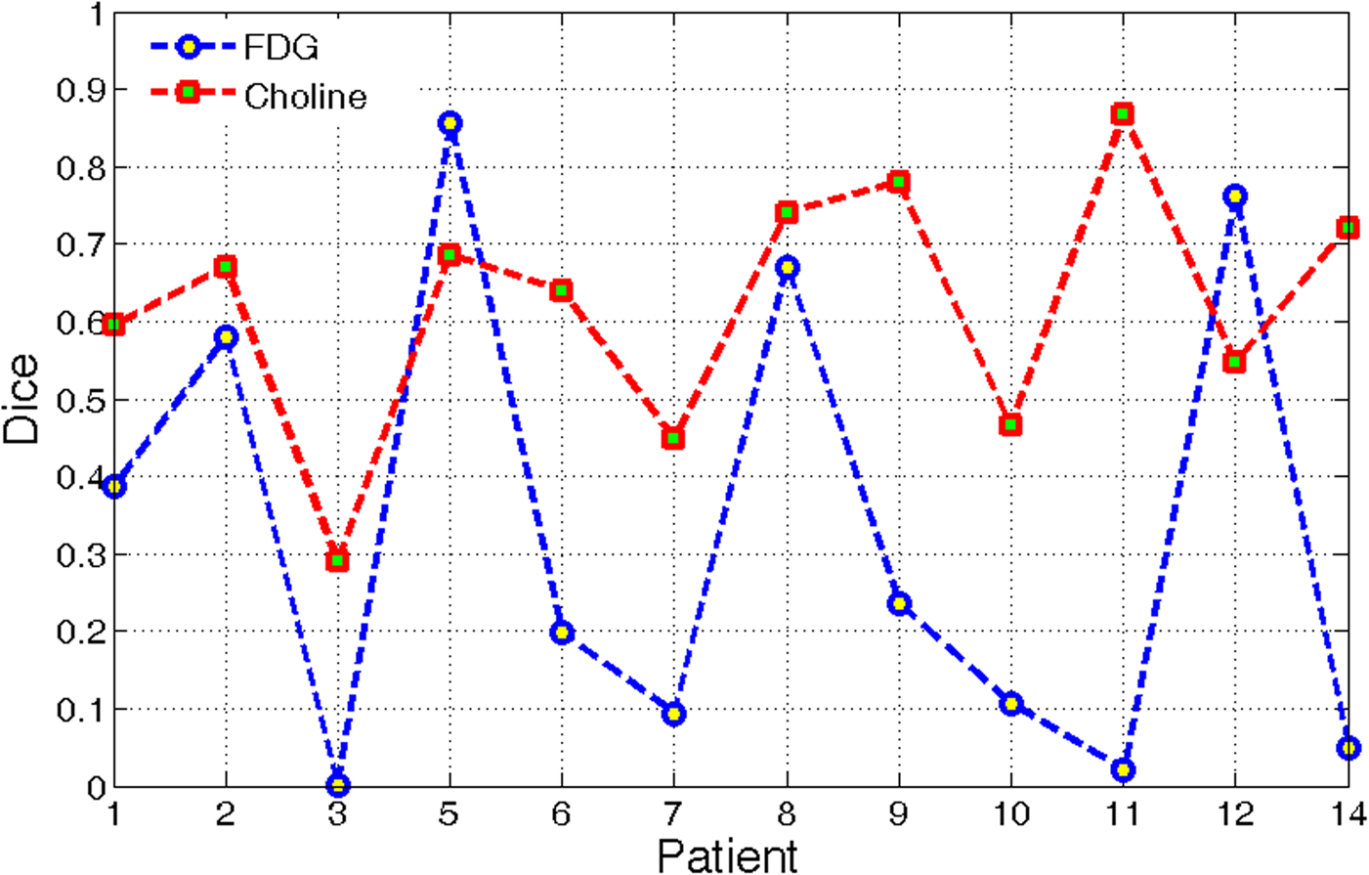
DSC of RG results using FDG and Choline PET/CT fusion.

The RG method obtained a higher mean score but lower standard deviation of DSC than the threshold method. RG method produced a more stable outcome; hence, the performances of this method using the two modalities were compared. DSCs of 12 patients were illustrated graphically in a polygon diagram [Fig 2].

The segmentation results of ^11^C-Choline PET exhibited a relatively higher average score than that of ^18^F-FDG PET, although two exceptions were presented. A significant difference was observed between the two types of PET volumes (p < 0.001). This phenomenon objectively revealed that ^11^C-Choline PET images provided a better distinction of NPC than ^18^F-FDG images.

## Discussion

In our previous study, qualitative visual assessment results suggested that the use of combined ^11^C-Choline PET/CT images could help prevent the risk of lesions that were not evidently found during diagnosis [11]. In the current study, a systematic evaluation on inter-observer and inter-modality variations of GTV contouring with the use of ^11^C-Choline and ^18^F-FDG NPC PET images for radiotherapy was presented.

The aforementioned studies have frequently demonstrated the reduction of the inter-observer variability when ^18^FDG-PET is incorporated in GTV delineation of non-small cell lung cancer [18]. Although the result is less consistent, this variation is also observed in patients with head and neck cancer [19]. In our investigation, inter-observer variation in the ^11^C-Choline group was lower than that in the ^18^F-FDG group. Oncologists obtain a consistent GTV when contouring ^11^C-Choline PET/CT images mainly because ^11^C-Choline PET images decrease the uncertainty of skull base or brain invasion of NPC, as observed in our previous assessment.

**Fig 3:**
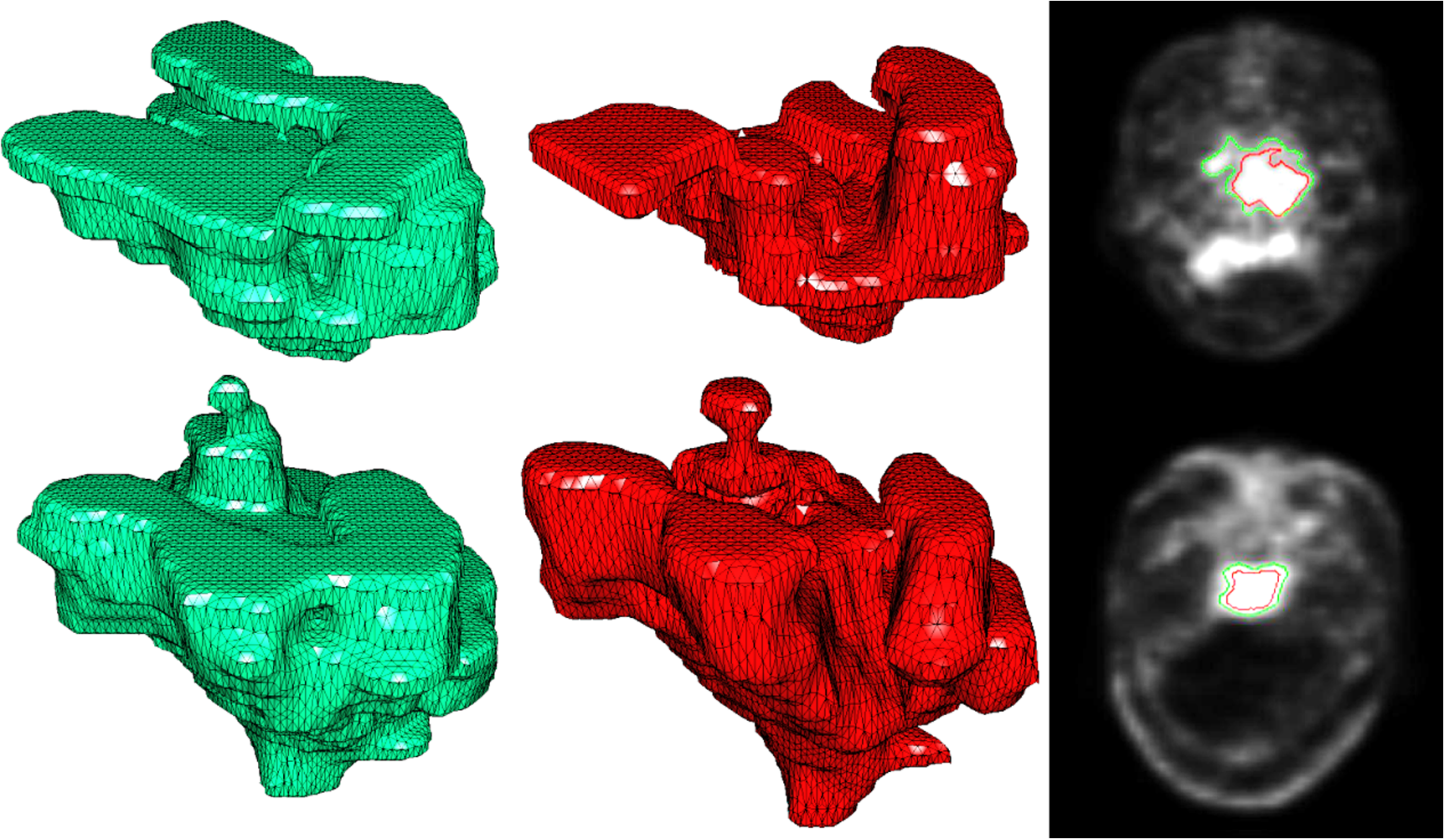
Solid rendering of manually contoured reference (green) and RG result (red). The first row shows the result of ^18^F-FDG; the second row shows the result of ^11^C-Choline. The DSCs of the two modalities were 67.06% and 74.11%, respectively. (^11^C-Choline scans were obtained 3 d after ^18^F-FDG).

Significant difference in inter-modality variations was observed between the ^18^F-FDG group and the ^11^C-Choline group. Oncologists likely contoured a larger region when they used ^11^C-Choline PET/CT fusions, and this result was observed mainly because of two factors. The objective reason is that the use of ^18^F-FDG PET for GTV definition usually underestimates tumor invasion [20]. Similar underestimation was also found in our assessment [Fig 3]. The subjective reason is that oncologists are conservative to the uncertainty of tumor invasion when ^18^FDG-PET images are used in diagnosis, particularly at sites where skull base or brain invasion occurs because tumors in these sites exhibit comparable SUV with the surrounding tissues. Hence, semi-automatic contouring method was introduced in our assessment. In this way, the influence of random factors can be effectively minimized and subjective mistakes can be avoided.

The shape of the ground truth and RG result of ^11^C-Choline PET were more similar to each other than that of ^18^F-FDG PET. The semi-automatic contouring method possibly underestimated the lesion when ^18^F-FDG PET was used, but semi-automatic contouring was prone to leak out at local areas adjacent to the encephalocoele. Computer-aided contouring methods reported in our investigation can be hardly admitted because the accuracy of the results was relatively low. These methods did not exhibit relevant results mainly because uptake values of lesions were overlapped with nearby normal tissues, low spatial resolution of PET images, and absence of anatomical information. With limited human interaction, the algorithm can hardly obtain accurate results in the whole image volume and is usually entrapped in over-segmentation (leakage) or under-segmentation (shrinkage). However, the segmentation results in some slices were comparable to that of manual contouring, particularly in some ^11^C-Choline slices presenting apparent skull invasion. This superiority provides treatment planners with a clinically viable starting point for tumor delineation and minimizes the inter-observer variability in radiotherapy planning.

The manual contouring result confirmed that the inter-observer variations were lower in the ^11^C-choline group. The semi-automatic contouring method being used also revealed that ^11^C-Choline PET images are beneficial to the lesion targeting. However, whether or not ^11^C-Choline PET images can be used to define GTV in radiotherapy planning remains unclear, because data of the treatment outcome obtained from this approach are insufficient. Moreover, the present study focused on GTV rather than on planned target volume (PTV). PTV is the volume at which a prescribed dosage is actually delivered, whereas GTV is the volume of gross disease. Hence, a study of PTV reproducibility under the same circumstances should be performed.

Clinical trials should first address the safety of this approach, and assessment of possible improvement of outcomes should be conducted. IEC (International Electro Technical Commission) phantoms, covering a range of spherical lesion sizes, contrast ratios, noise levels and voxel sizes, should be used to figure out the mechanism of significant difference in inter-modality variations. In addition, the guidelines for GTV contouring using different PET images should be assessed on realistic non-uniform and non-spherical volumes simulated from patient lesions.

## Conclusions

^11^C-Choline PET/CT images could be introduced as an important complementary tool to decrease inter-observer variation in GTVs obtained for NPC. ^11^C-Choline PET/CT images provided larger GTV volumes than FDG images. Phantom simulations and clinical trials should be conducted to prove the possible improvement of the treatment outcome.
